# Identification of a mouse *Lactobacillus johnsonii *strain with deconjugase activity against the FXR antagonist T-β-MCA

**DOI:** 10.1371/journal.pone.0183564

**Published:** 2017-09-14

**Authors:** Michael DiMarzio, Brigida Rusconi, Neela H. Yennawar, Mark Eppinger, Andrew D. Patterson, Edward G. Dudley

**Affiliations:** 1 Department of Food Science, The Pennsylvania State University, University Park, PA, United States of America; 2 Department of Biology and South Texas Center for Emerging Infectious Diseases, University of Texas at San Antonio, San Antonio, TX, United States of America; 3 Huck Institutes of the Life Sciences, The Pennsylvania State University, University Park, PA, United States of America; 4 Department of Veterinary and Biomedical Sciences, The Pennsylvania State University, University Park, PA, United States of America; Instituto de Agroquimica y Tecnologia de Alimentos, SPAIN

## Abstract

Bile salt hydrolase (BSH) activity against the bile acid tauro-beta-muricholic acid (T-β-MCA) was recently reported to mediate host bile acid, glucose, and lipid homeostasis via the farnesoid X receptor (FXR) signaling pathway. An earlier study correlated decreased *Lactobacillus* abundance in the cecum with increased concentrations of intestinal T-β-MCA, an FXR antagonist. While several studies have characterized BSHs in lactobacilli, deconjugation of T-β-MCA remains poorly characterized among members of this genus, and therefore it was unclear what strain(s) were responsible for this activity. Here, a strain of *L*. *johnsonii* with robust BSH activity against T-β-MCA *in vitro* was isolated from the cecum of a C57BL/6J mouse. A screening assay performed on a collection of 14 *Lactobacillus* strains from nine different species identified BSH substrate specificity for T-β-MCA only in two of three *L*. *johnsonii* strains. Genomic analysis of the two strains with this BSH activity revealed the presence of three *bsh* genes that are homologous to *bsh* genes in the previously sequenced human-associated strain *L*. *johnsonii* NCC533. Heterologous expression of several *bsh* genes in *E*. *coli* followed by enzymatic assays revealed broad differences in substrate specificity even among closely related *bsh* homologs, and suggests that the phylogeny of these enzymes does not closely correlate with substrate specificity. Predictive modeling allowed us to propose a potential mechanism driving differences in BSH activity for T-β-MCA in these homologs. Our data suggests that *L*. *johnsonii* regulates T-β-MCA levels in the mouse intestinal environment, and that this species may play a central role in FXR signaling in the mouse.

## Introduction

The gut microbiota has received increasing attention throughout the last decade for its role in human and animal health [1,2]. For example, metagenomic studies and functional analyses including metabolomics have identified a potential correlation between the composition of the gut microbiota and the development of obesity [3–5]. A mechanistic understanding of bacterial mediated metabolic regulation is only just beginning to emerge.

Several recent studies have elucidated a metabolic control system in the intestine based on interactions between the gut microbiota, bile acids, and the farnesoid X receptor (FXR) [6–8]. Bile acids are conjugated to either taurine or glycine by mice and humans respectively in the liver, and are transported through the bile duct to the small intestine. In the intestine, microbial bile salt hydrolases (BSHs) produced by several genera including *Lactobacillus*, *Bifidobacterium*, *Bacteroides*, and *Clostridium* [9,10] facilitate the deconjugation of the amino acid, effectively removing the bile acid from enterohepatic recirculation [11]. BSHs facilitate intestinal colonization of these organisms, potentially through liberation of amino acids or modulation of bacterial membrane fluidity [11].

FXR is a ligand activated transcription factor which primarily regulates bile acid synthesis from cholesterol in the liver and also affects glucose and lipid metabolism [12]. Most endogenous bile acids bind and activate FXR, however, tauro-beta-muricholic acid (T-β-MCA) has been shown to uniquely antagonize FXR, making it an important counterbalance for maintaining metabolic homeostasis [6,7]. Interactions between the gut microbiota and the host bile acid pool, particularly T-β-MCA, influence FXR signaling and ultimately host metabolism, and appear to be driven in large part by *Lactobacillus* [6,7,13]. Although T-β-MCA is not a primary bile acid in humans, studying its role in mice broadens our understanding of BSH expression by the gut microflora and its impact on host health.

Li *et al*. [7] previously reported that feeding the antioxidant tempol to mice significantly lowered rates of weight gain for mice on a high fat diet, which correlated with reduced *Lactobacillus* populations and corresponding increases in T-β-MCA concentrations [7]. No attempt was made to identify a direct link between *Lactobacillus* abundance and BSH activity against T-β-MCA. Here, we hypothesized that the intestines of C57BL/6J mice used in earlier experiments are colonized by a *Lactobacillus* strain that deconjugates T-β-MCA. We also screened a wider collection of *Lactobacillus* for BSH activity against T-β-MCA. Lastly, in an effort to understand the factors affecting BSH substrate specificity, we modeled critical interactions within the substrate binding pocket of BSHs exhibiting activity against T-β-MCA. Our work suggests that *Lactobacillus* strains in the mouse are important mediators of metabolism through their interactions with the potent FXR antagonist T-β-MCA.

## Results

### Isolation of mouse intestinal lactobacilli

Using 16S rRNA analysis, *Li* et al. [7] observed a reduction in the relative proportion of *Lactobacillus* spp. within the cecum of mice fed the antioxidant tempol, and these mice were leaner and had increased levels of intestinal T-β-MCA. No attempt was made however to isolate any lactobacilli for detailed analysis. We therefore hypothesized that the cecal contents from C57BL/6J mice used in the earlier study would be colonized by a *Lactobacillus* spp. with deconjugation activity towards T-β-MCA. Twenty isolates obtained were all identified as *L*. *johnsonii* based on 16S rRNA sequencing using universal bacterial primers [14]. Comparison of growth rates and acidification of MRS media revealed no noticeable phenotypic differences among the isolates (data not shown), and therefore a single, representative isolate, designated LB1, was selected for further characterization.

### *Lactobacillus* BSH activity against T-β-MCA

BSH activity against the potent FXR antagonist T-β-MCA has been shown to regulate metabolism, and is likely dependent on the strain specific composition of the gut microbiota [6,7,13]. Therefore, we determined if LB1 is capable of deconjugating T-β-MCA *in vitro*. Cultures and cell lysates of LB1 spiked with T-β-MCA were analyzed by ultra-performance liquid chromatography coupled with electrospray ionization quadrupole time-of-flight mass spectrometry (UPLC-ESI-QTOFMS) and found to have undergone nearly complete reductions in the peak area associated with T-β-MCA (m/z 514.2844) with corresponding increases in the peak area associated with β-MCA (m/z 407.2803) (Figure A in S1 File) These results indicate that LB1 exhibits BSH activity towards T-β-MCA (Table 1) and suggest that changes in intestinal LB1 populations have the potential to mediate FXR signaling.

**Table 1:** BSH activity against T-β-MCA^1^ in *Lactobacillus* strains.

*Lactobacillus* strain ID	MRS^2^	MRS + 0.1% porcine bile
*L*. *acidophilus* NCFM	**-**	**-**
*L*. *acidophilus* La5	**-**	**-**
*L*. *plantarum* WCFS1	**-**	**-**
*L*. *plantarum* Lp 39 (ATCC 14937)	**-**	**-**
*L*. *reuteri* SD2112 (ATCC 55730)	**-**	**-**
*L*. *reuteri* MM4-1A	**-**	**-**
*L*. *gasseri* DSM 20243 (ATCC 33323)	**-**	**-**
*L*. *johnsonii* VPI 7960 (ATCC 33200)	**-**	**-**
*L*. *johnsonii* LB1	**+**^3^	**+**
*L*. *johnsonii* NCK88 (ATCC 11506)	**+**	**+**
*L*. *brevis* 118–8 (ATCC 367)	**-**	**-**
*L*. *fermentum* B1 28 (ATCC 14931)	**-**	**-**
*L*. *salivarius* subsp. *salivarius* HO66 (ATCC 11741)	**-**	**-**
*L*. *rhamnosus* GG (ATCC 53103)	**-**	**-**

^1^ tauro-beta-muricholic acid

^2^ MRS: deMan, Regosa, Sharpe broth

^3^Positive activity is defined as a mean 20% reduction in T-β-MCA concentration from three independent assays.

Next, we used this assay to screen a collection of *Lactobacillus* strains in order to identify additional strains with BSH activity against T-β-MCA. We selected *Lactobacillus* strains which are fully sequenced, encode at least one BSH gene, and are publically available through the American Type Culture Collection (ATCC). Each strain was incubated in both MRS and MRS containing 0.1% porcine bile. Two growth conditions were used in order to determine if activity was inducible. Only LB1 and NCK88, both strains of *L*. *johnsonii*, were capable of deconjugating T-β-MCA (Table 1). *L*. *johnsonii* ATCC33200 did not exhibit any activity against T-β-MCA, a finding that reinforces an extensive body of evidence indicating that BSH activity is strain dependent in *Lactobacillus* [11]. None of the additional 11 *Lactobacillus* strains we screened exhibited any detectable activity against T-β-MCA regardless of whether or not they were pre-incubated in media containing bile. These results led us to further investigate BSH activity in *L*. *johnsonii*.

### Genomic comparison of *L*. *johnsonii* strains

**Fig 1:**
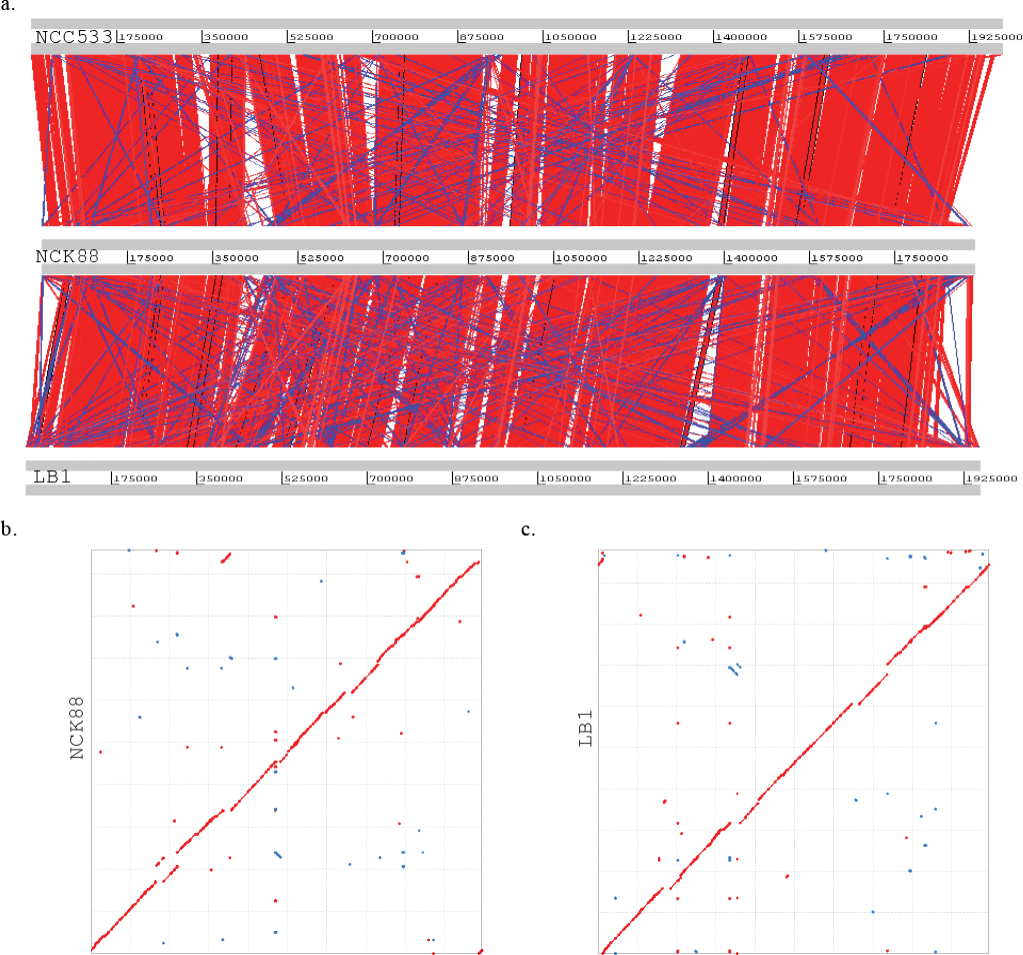
Visual comparisons of *L*. *johnsonii* NCK88 and LB1 draft genomes to the reference strain NCC533. (a) WebACT visualization of genome similarity of NCK88 and LB1 to NCC533 based on BlastN analysis with a minimum match size of 100 nucleotides. Red bars indicate matches in the same orientation, and blue bars indicate matches in the reverse orientation. (b and c) Dot plots representing the maximum unique matches (MUMs) of the six frame amino acid translations of LB1(b) and NCK88 (c) draft genomes relative to NCC533. Forward MUMs are plotted as red lines/dots while reverse MUMs are plotted as blue lines/dots. A line of dots with slope of 1 represents an undisturbed segment of conservation between the two sequences and a line with a slope of -1 represents an inverted segment of conservation between the two sequences.

To gain insight into the genome organization and genetic basis of activity against T-β-MCA in LB1 or NCK88, we sequenced and assembled draft genomes of each strain in order to compare them to *L*. *johnsonii* NCC533, an *L*. *johnsonii* strain with a fully completed and closed genome [15]. A visual comparison of LB1 and NCK88 contigs mapped against the closed reference *L*. *johnsonii* NCC533 indicated that all three strains have a similar genome architecture, size, and GC content (Fig 1A). Dot plot comparisons of translated open reading frames also revealed strong conservation in overall proteome similarity and syntenic gene organization, with little evidence of large scale rearrangements, such as inversions or translocations (Fig 1B and 1C).

**Fig 2:**
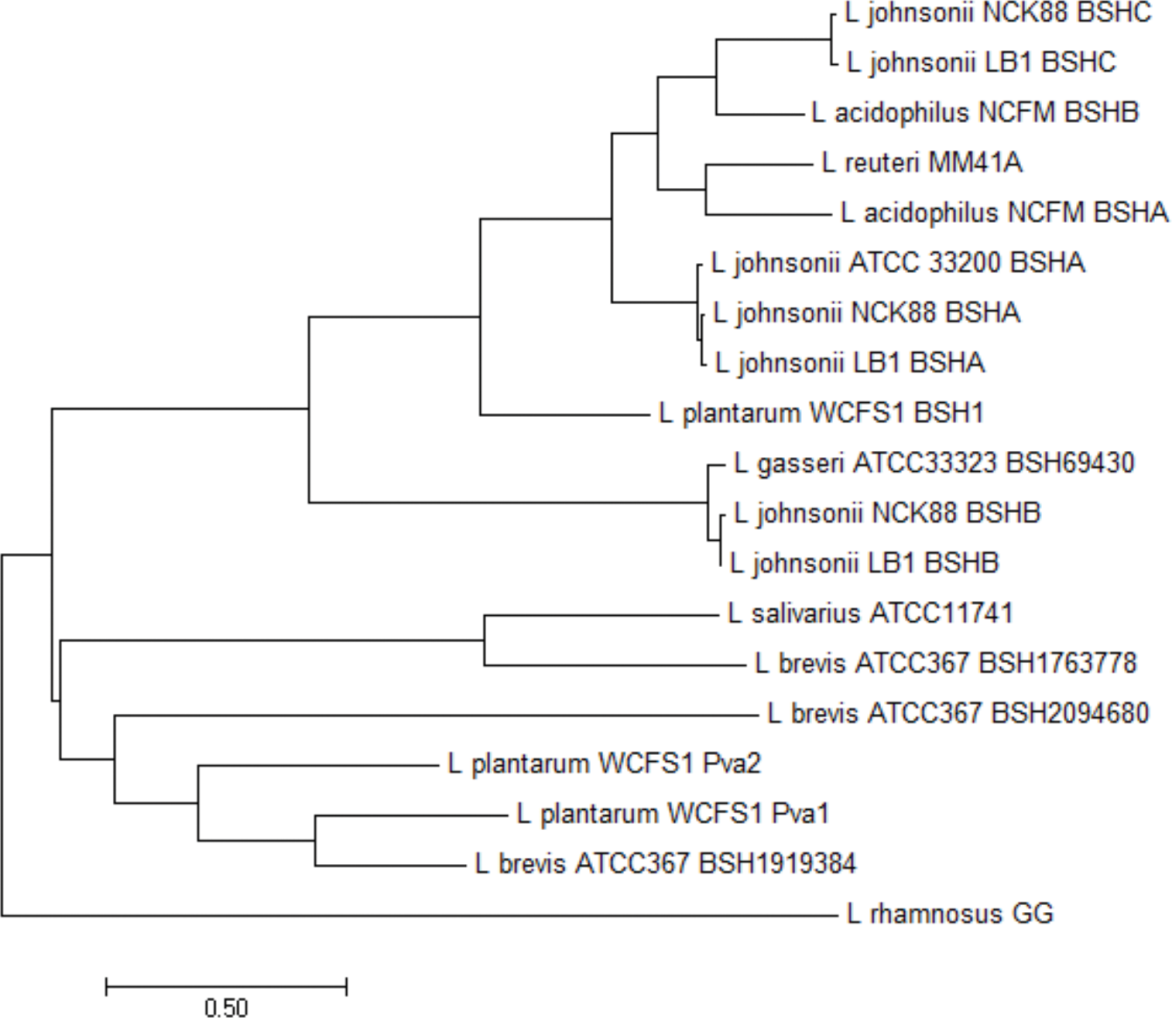
Phylogenetic analysis of *Lactobacillus* BSH sequences by maximum likelihood method. The evolutionary history of BSHs encoded by *Lactobacillus* strains screened in our *in vitro* assay was inferred by using the Maximum Likelihood method based on the Le Gascuel 2008 model [17]. The tree with the highest log likelihood (-8040.5390) is shown. Initial trees for the heuristic search were obtained automatically by applying Neighbor-Join and BioNJ algorithms to a matrix of pairwise distances estimated using a JTT model, and then selecting the topology with superior log likelihood value. A discrete Gamma distribution was used to model evolutionary rate differences among sites (3 categories (+*G*, parameter = 2.2994)). The rate variation model allowed for some sites to be evolutionarily invariable ([+*I*], 4.7893% sites). The tree is drawn to scale, with branch lengths measured in the number of substitutions per site. The analysis involved 19 amino acid sequences. All positions containing gaps and missing data were eliminated. There were a total of 297 positions in the final dataset. Evolutionary analyses were conducted in MEGA6 [18].

Next, we used BLAST [16] to identify BSH loci within the draft genomes of LB1 and NCK88 that are similar to those previously described in *L*. *johnsonii* NCC533 [15]. LB1 and NCK88 each contain three BSH loci in the same relative genomic locations, that we designated BSHA, BSHB, and BSHC, which share 98.5, 99.1, and 98.2% amino acid identity to each other and are homologous to LJ1412, LJ0056, and LJ1147 in *L*. *johnsonii* NCC533, respectively (Figures B and C and D in S1 File). A subsequent protein BLAST (BLASTp) [16] comparison of all three *L*. *johnsonii* BSH homologs against the non-redundant NCBI database failed to identify any of these in other species. Hierarchical clustering and phylogenetic analysis of the BSHs from strains in Table 1 was used to assess the relatedness of BSHs among different *Lactobacillus* spp (Fig 2). BSHA in LB1 and NCK88 are nearly identical and share a distinct branch with the BSHA homolog identified in *L*. *johnsonii* ATCC33200. BSHB in LB1 and NCK88 are closely related to the BSH identified in *L*. *gasseri* ATCC33323, and BSHC from both strains are related to *L*. *acidophilus* BSHB. Notably, closely related BSHs likely exhibit differences in substrate specificity for T-β-MCA, since *L*. *acidophilus* NCFM, *L*. *gasseri* ATCC33323, and *L*. *johnsonii* ATCC33200 did not display any activity against T-β-MCA in our *in vitro* assay (Table 1).

### Comparison of BSH substrate specificity

In order to identify which BSH genes from *L*. *johnsonii* had specificity towards T-β-MCA, we cloned each BSH gene from strains LB1, NCK88, and ATCC33200 downstream of a constitutive promoter encoded on pSF-OXB12 and transformed these constructs into *E*. *coli*. BSHA from *L*. *johnsonii* ATCC33200 and LB1 did not exhibit any activity against T-β-MCA (Table 2). BSHB from LB1 and NCK88, which are 98.7% identical (Figure B in S1 File), completely deconjugated the full range of substrates. Interestingly, LB1 BSHC also effectively deconjugated all of the bile acids, but NCK88 BSHC was not active against any tauro-conjugated substrates, including T-β-MCA—these enzymes only differ by six amino acids (Figure D in S1 File).

**Table 2:** Substrate specificity of heterologous expressed bile salt hydrolases in *Escherichia coli*.

	T-β-MCA^1^	TCA	TCDCA	TDCA	GCA	GCDCA
*L*. *johnsonii* LB1 BSHA	**-**	**-**	**-**	**-**	**-**	**-**
*L*. *johnsonii* LB1 BSHB	**+**^2^	**+**	**+**	**+**	**+**	**+**
*L*. *johnsonii* LB1 BSHC	**+**	**+**	**+**	**+**	**+**	**+**
*L*. *johnsonii* NCK88 BSHB	**+**	**+**	**+**	**+**	**+**	**+**
*L*. *johnsonii* NCK88 BSHC	**-**	**-**	**-**	**-**	**+**	**+**
*L*. *johnsonii* ATCC 33200 BSHA	**-**	**-**	**-**	**-**	**-**	**-**

^1^ Bile acids are: T-β-MCA, tauro-beta-muricholic acid; TCA, taurocholic acid; TCDCA, taurochenodeoxycholic acid; TDCA, taurodeoxycholic acid; GCA, glycocholic acid; GCDCA, glycochenodeoxycholic acid.

^2^ Positive activity is defined as a mean 20% reduction in substrate concentration from three independent assays.

### Modeling BSH substrate interactions with T-β-MCA

**Fig 3:**
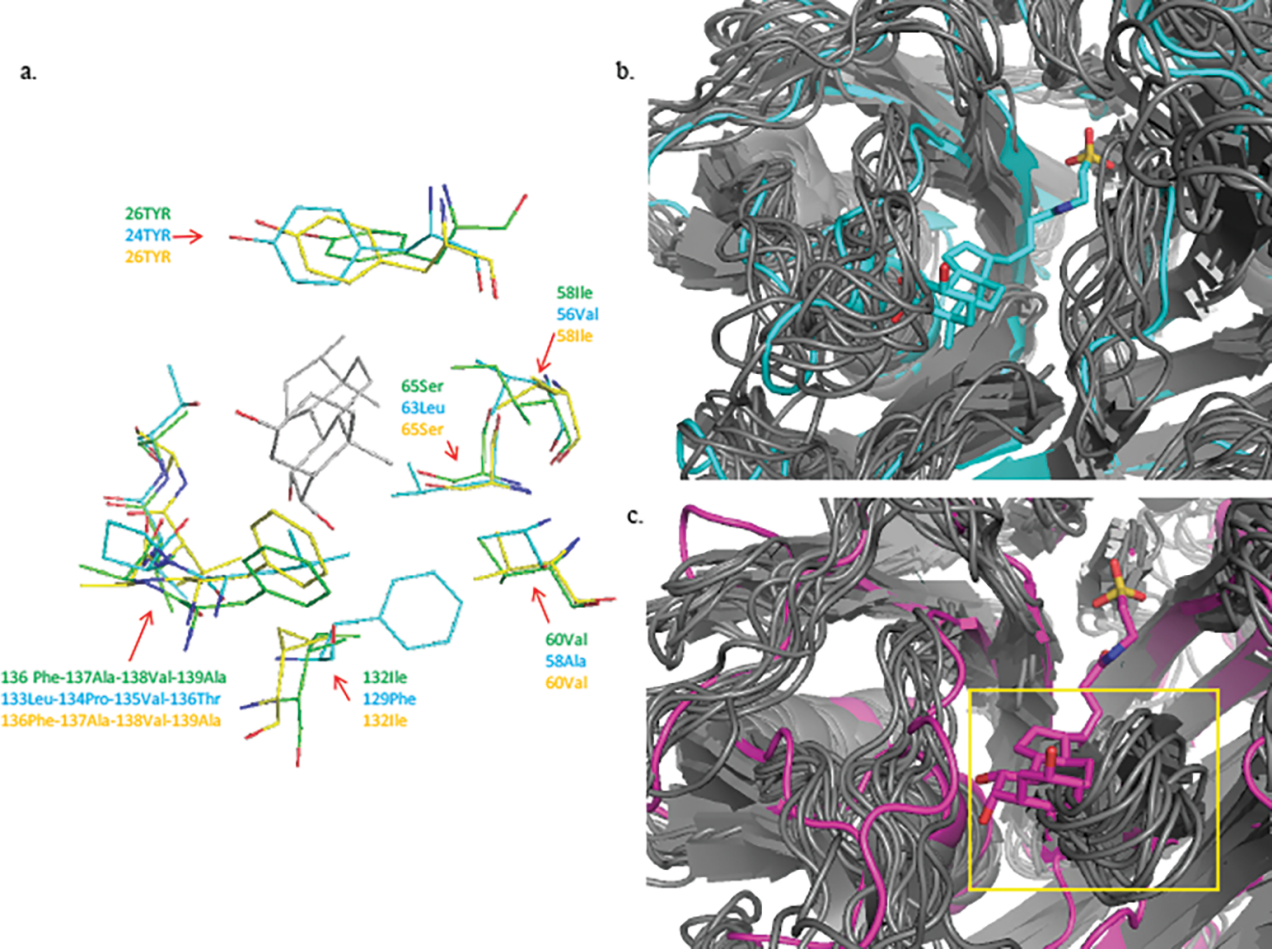
Visualizations of model differences in BSH substrate interactions with T-β-MCA. (a) Depiction of residue side chains within a 4Å radius of the T-β-MCA ring structure in the substrate binding pocket of enzymes with activity against T-β-MCA. LB1 BSHC is in blue, LB1 BSHB is in green, NCK88 BSHB is in yellow, and T-β-MCA is in grey. Stick depictions of T-β-MCA and amino acid side chains indicate oxygen atoms in red, and nitrogen atoms in dark blue. Hydrogens have been removed to improve visual clarity. (b and c) Cartoon comparisons of LB1 BSHC (b) and NCK88 BSHC (c) with T-β-MCA in the binding pocket. YASSARA models energetically minimized to T-β-MCA are depicted in blue (LB1 BSHC) and magenta (NCK88 BSHC), and the range of loop movements for each model based on CABS-flex simulations is depicted in grey. Occlusion of the substrate binding pocket by the loop structure in NCK88 BSHC is indicated in a yellow box in (c).

Lastly, we used predictive modelling to generate hypotheses concerning the limited substrate specificity observed for the BSHA homologs and the differences in substrate specificity observed between the two BSHC homologs. Previous studies have shown that the active site of BSHs is comprised of two regions which interact with the conjugated bile acid and influence substrate specificity [19,20]. The first is a snug hydrophobic pocket lined by a number of nonpolar residues that stabilizes the steroid moeity of the substrate through van der Waals interactions and the second is a positively charged surface where the negatively charged SO_3_ modification of the taurine or glycine anchors. We utilized predictive modeling to computationally identify and compare relevant structural differences among *L*. *johnsonii* BSHs which might contribute to determining substrate specificity, particularly for T-β-MCA. Our calculated models are consistent with the crystal structure of the well characterized conjugated BSH from *Clostridium perfringens* [19], and did not change when models included the different bile acids screened earlier (data not shown). An analysis of distance measurements suggests that critical arginine, asparagine, and N-terminal cysteine residues are capable of hydrogen bonding with taurine in the active site in all of the *L*. *johnsonii* BSHs (Table A in S1 File) [19]. Hydrophobic interactions between amino acid residues in the substrate binding pocket and the ring structure of β-MCA are generally well conserved among the BSHs exhibiting activity against T-β-MCA (Fig 3A, Table A in S1 File). BSHA from strains LB1 and ATCC33200 both have unresolvable steric clashes between the aromatic side chain of Phe_66_ and the steroid moiety of all of the bile acid substrates, providing a possible explanation for their lack of experimental activity (Table 2). Interestingly, BSHC from LB1 and NCK88, which share 98.2% amino acid identity but differ in their substrate specificity for T-β-MCA, exhibit noticeable differences in the positions of the loop structures defining the opening to the substrate binding pocket (Fig 3B and 3C). This pocket spans 20.1 Å in NCK88 BSHC, and only 15.2 Å in LB1 BSHC based on the position of the loops in our original energy minimized models. To further investigate the potential relevance of the loop structures lining the substrate binding pocket in these enzymes, we used CABS-flex to simulate protein structure fluctuations and assess the range of motion for these loops [21]. The dynamic models revealed that the range of movement in the loop structure comprised of Val_57_-Ala_58_-Asn_59_-Asp_60_-Tyr_61_-Pro_62_-Leu_63_ occludes the steroid binding pocket in NCK88 BSHC and likely contributes to its more limited substrate specificity (Fig 3B and 3C).

## Discussion

Li *et al*.[7] reported that the antioxidant tempol decreased *Lactobacillus* populations in the cecum which was proposed to be responsible for increases in intestinal T-β-MCA concentrations and decreases in FXR signaling. However, this study did not directly attribute BSH activity against T-β-MCA to any *Lactobacillus* strain [7]. Importantly, the observed changes in *Lactobacillus* populations were part of a broader shift in the overall composition of the gut microbiota, and BSHs have been identified in every representative lineage throughout the microbial community [22]. Therefore, it remained unclear if more specific changes in *Lactobacillus* populations were capable of influencing compositional changes in host bile acid composition with respect to T-β-MCA. Our results provide evidence that a *L*. *johnsonii* strain, LB1, exhibits BSH activity against T-β-MCA, a critical mediator of FXR signaling. In one animal in our C57BL/6J colony, *L*. *johnsonii* appeared to be the predominant lactobacilli colonizing the intestinal tract, which is consistent with findings of others [23]. Additional work is required to determine whether this was also the dominant *Lactobacillus* strain in other mice in our colony.

Genomic comparison of *L*. *johnsonii* LB1 isolated in this study, and NCK88, which is of unknown origin, to the human strain NCC533 revealed a high degree of similarity among the strains. We identified three independent homologs of *bsh* in all three strains, and one of the three homologs was also identified in *L*. *johnsonii* ATCC33200. Phylogenetic comparison of these BSHs among a broader collection of BSHs on an amino acid level revealed close evolutionary relationships among *L*. *johnsonii* BSHB and the BSH found in *L*. *gasseri* ATCC33323 as well as *L*. *johnsonii* BSHC and *L*. *acidophilus* NCFM BSHB. *L*. *johnsonii* BSHA meanwhile appears more distantly related to BSHs from other strains, which perhaps reflects its lack of BSH activity. These results largely agree with a report of predicted protein similarity among these species and suggest that BSHs are likely following the proposed stepwise vertical evolution within the *L*. *acidophilus* group [24]. Indeed, none of the *L*. *johnsonii* BSH homologs were identified in any other species. While useful for identifying homology and delineating evolutionary relationships among BSHs, it is also apparent from our screening results that hierarchical clustering and phylogenetic analysis of BSH amino acid sequences does not closely parallel the associated bile acid substrate specificities of these enzymes. For example, despite encoding BSHs closely related to those found in *L*. *johnsonii*, *L*. *acidophilus* NCFM and *L*. *gasseri* ATCC33323 did not exhibit any activity against T-β-MCA. Additionally, BSHC in *L*. *johnsonii* LB1 and NCK88 are indistinguishable phylogenetically, but exhibit broad differences in substrate specificity.

Conjugated muricholic acids are capable of acting as FXR antagonists, making them important potential targets for BSHs in the intestine [6,25]. The role of T-β-MCA in the regulation of FXR signaling is now firmly established in mice, and it has been identified as part of a complex pool of bile acids found in humans [6,7,26–29]. BSH activity has historically been considered a desirable trait in *Lactobacillus* probiotics for bile tolerance as well as maintenance of host cholesterol levels, raising the question of whether any commercially available probiotics are capable of altering intestinal T-β-MCA concentrations [11,30]. Previous characterizations of BSH activity in probiotics have revealed considerable diversity in substrate specificity for glyco- and tauro-conjugates of CA, DCA, and CDCA, but activity against T-β-MCA has rarely been studied [31–34]. Several bacterial strains isolated from a healthy human microbiota have been reported to deconjugate T-β-MCA, including *Bacteroides vulgatus*, *Clostridium ramosum*, *Bifidobacterium longum*, *Peptostreptococcus productus* and *L*. *gasseri*, but the genes encoding these enzymes were never identified [35–37]. Likewise, the complete mouse gut microbiota has been shown to exhibit BSH activity against T-β-MCA, and recently, Joyce *et al*. identified a BSH from the strain *L*. *salivarious* JCM1046 with activity against a broad range of mouse bile acids, including T-β-MCA [6,13]. Since *L*. *johnsonii* is closely related to many strains of *Lactobacillus* with well characterized BSH activity against tauro-conjugates of CA, DCA, and CDCA, we were surprised that none of the other strains we considered exhibited detectable activity against T-β-MCA. The prevalence of BSHs with activity against T-β-MCA is not clear from our limited study, but our results raise an important ecological question regarding why closely related bacterial species have acquired BSHs with distinct substrate specificities against T-β-MCA. These results also suggest that species and strain level compositional changes within the gut microbiota may have important consequences for host bile acid metabolism. The *in vivo* significance of this activity towards T-β-MCA needs further investigation given that the same enzyme is also active against FXR agonists CDCA and TCA.

A large number of BSH genes have been identified through metagenomics but have never been characterized *in vitro*, making it difficult to understand how shifts in the gut microbial community might affect host bile acid composition [22]. This would be particularly useful for mining clinical data relating to probiotics and obesity for added insights into the underlying role of BSHs, and would ultimately inform the development of next generation probiotics for controlling metabolism [38,39]. Data from our predictive modeling experiments generally aligns with the results of our *in vitro* screening assay, and provide new insights into the basis for BSH substrate specificity. Substrate specificity is linked to bile acid interactions with both the amino acid binding region and the hydrophobic steroid binding pocket within the active site of the enzyme, and thus activity is likely lower if either of the surfaces is not optimally matched. Ideally, the hydrophobic pocket should contour the steroid rings with no steric hindrance from protein residues and also should not be too large to have favorable van der Waals interactions. Interactions at the amino end also need to be suitable for hydrogen bonding with the SO_3_ group and the protonation state of the cysteine side chain must be optimal to facilitate cleavage of the bond. Indeed, our models implicated unfavorable side chain positioning in the hydrophobic pocket in the lack of activity we observed for *L*. *johnsonii* BHSA, but we were unable to identify differences in critical hydrogen bond interactions at the amino end among the active enzymes (Table 2). Although both enzymes appear capable of forming critical hydrogen bonds between Cys_2,_ Asp_19_, and Asn_79_ residues and taurine, NCK88 BSHC did not exhibit activity against any tauro-conjugated bile acids in our assay, so it is likely that differences in the configuration of substrate binding pocket of the active site compared to LB1 BSHC are also contributing to its narrower substrate specificity (Table A in S1 File). Notably, a crystal structure depicting the intact bile acid in the binding pocket of a BSH is not available, and taurine is thought to only partially occupy the space, so our ability to effectively model these interactions is limited [19]. Mutated residues Ser/Asn_108_, Thr/Pro_122_ and Thr/Ala_289_ (residues in NCK88/LB1) in a 12 Å sphere of the active site appear to perturb the substrate binding dynamics in the active site region (Figure C in S1 File). We suggest that the relative position of the loop structures defining the edge of the substrate binding pocket and their inherent flexibility may contribute to the differences in substrate specificity, as an increased range of loop movement in NCK88 BSHC hinders substrate binding. Such long range interactions are quite common in evolutionary related proteins as seen by a statistical coupling analysis of residue networks [40].

Our UPLC-ESI-QTOFMS-based screening assay for activity against T-β-MCA clearly and effectively identified two strains of *L*. *johnsonii* and improves on existing BSH substrate specificity screening methods such as the costly and subjective agar plate precipitation assay or an assay based on incubation in extracted mouse bile [11,13]. Admittedly, quantification of specific enzymatic activity is not possible with our experimental design, as we aimed to identify strains of *Lactobacillus* with clear potential to alter T-β-MCA concentrations *in vivo*. We were careful to consider the potential for inducible BSH expression in our screen, but it is still possible that factors such as growth state, bile acid transport, and culture conditions affect apparent BSH activity and may have caused us to overlook strains with low levels of T-β-MCA activity.

This work directly characterized T-β-MCA substrate specificity in strains of *Lactobacillus* using a clear and cost effective screening assay, and our results indicate that activity against T-β-MCA cannot be easily inferred from previously characterized activity against common tauro-conjugated bile acids. Our data suggests that differences in BSH substrate specificity may manifest even among highly homologous BSHs. Ultimately, these results lay the groundwork for future *in vivo* experiments with *L*. *johnsonii* LB1 and NCK88 aimed at exploiting FXR mediated metabolic signaling by altering intestinal T-β-MCA concentrations.

## Materials and methods

### Bacterial strains and growth conditions

*L*. *johnsonii* NCK88, *L*. *acidophilus* NCFM and *L*. *plantarum* WCFS1 were generously provided by Dr. Todd Klaenhammer, *L*. *johnsonii* LB1 was isolated directly from mouse cecal contents, and all other *Lactobacillus* strains were purchased from the American Type Culture Collection (ATCC). Cultures were inoculated in sterile de Man, Rogosa, Sharp (MRS) [41] broth from 10% frozen glycerol stocks and incubated anaerobically in an atmosphere composed of 85% N, 10% CO_2_, and 5% H_2_ at 37°C for two consecutive passages. *E*. *coli* C600 and *E*. *coli* DH5α were inoculated from 10% frozen glycerol stocks in sterile Lysogeny Broth (LB) and incubated aerobically at 37°C with 300 RPM shaking agitation. Transgenic *E*. *coli* C600 strains harboring pSF-OXB12:BSH plasmids were inoculated in sterile LB supplemented with 50 μg/mL kanamycin and incubated at 37°C with 300 RPM shaking agitation for two consecutive passages.

### Enumeration and isolation of *Lactobacillus*

Cecal contents were harvested from healthy 6 week old male C57BL/6J mice fed ad libitum using standard NIH 31 chow, and diluted sufficiently in sterile PBS pH 7.4 to achieve isolated colonies on agar plates. Mice were sacrificed after carbon dioxide asphyxiation followed by cervical dislocation. This protocol was reviewed and approved by the Penn State Institutional Animal Care and Use Committee (Approval number 45503). Dilutions were plated in duplicate on BBL *Lactobacillus* selective (LBS) [42] agar and incubated anaerobically in an atmosphere composed of 85% N, 10% CO_2_, and 5% H_2_ for 48 hours at 37°C for colony counting. Twenty individual colonies were randomly selected and streaked onto MRS agar [41] for additional purification and designated as strains LB1- LB20. Isolated colonies were inoculated in MRS broth, grown anaerobically at 37°C overnight, and stored for future work at -80°C in 10% glycerol. Each of the 20 isolates was characterized by 16S rRNA sequencing as described below, and by measuring the acidification rate during anaerobic growth in MRS broth at 37 ^o^C.

### Identification and characterization of *Lactobacillus*

Isolated colonies were scraped into a PCR tube and microwaved for 5 mins to rupture the cells. PCR was performed using primers specific to *Lactobacillus* 16S rRNA using conditions described by Byun *et al*. [14]. Reaction products were visualized on a 2.0% agarose gel with SYBR-SAFE dye (Applied Biosystems) and treated with 10 units of exonuclease I (New England Biolabs), and 1 unit of Antarctic phosphatase (New England Biolabs) at 37°C for 45 minutes to remove unincorporated primers and dNTPs, then at 85°C for 15 minutes to inactivate the enzymes. Purified PCR amplicons were sequenced by the Penn State Genomics Core Facility by Sanger sequencing on an Applied Biosystems 3730XL. Individual sequences were assembled using the DNASTAR Lasergene 12 software suite (DNASTAR Inc.) and identified using NCBI’s BLAST database [43]. All isolates were also characterized phenotypically, measuring growth and acidification rate in MRS broth. Each isolate was inoculated in sterile MRS broth and incubated anaerobically at 37°C for 24 hours. OD_600_ and pH measurements were taken every four hours. All isolates were determined to be *L*. *johnsonii* by 16S rRNA analysis, and all grew in and acidified MRS at indistinguishable rates. Thus, one isolate was chosen, designated LB1, and used for further experiments.

### Genomic DNA sequencing

*L*. *johnsonii* strains LB1 and NCK88, a well-studied *L*. *johnsonii* isolate, were each inoculated from frozen glycerol stocks into sterile MRS and incubated anaerobically at 37°C overnight. Cells were precipitated by centrifugation and genomic DNA was extracted using Promega’s Wizard DNA kit. Whole genome sequencing was performed by the Penn State Genomics Core Facility using an Illumina MiSeq instrument with 300 bp paired end reads. Sequencing data was assembled *de novo* with DNASTAR’s SeqMan NGen (DNASTAR Inc.), and contigs were mapped against the reference *L*. *johnsonii* NCC533 genome with progressiveMauve [44,45]. Genomes were compared with WebACT and dotplots of translated open reading frames were generated with PROmer in Galaxy [46–50]. Sequence data is available under BioProject ID PRJNA315676.

### BSH sequence comparisons

BSH sequences from each strain in our *Lactobacillus* collection with publically available genomes were identified using NCBI’s Gene database. BSH genes from the fully sequenced and annotated strain *L*. *johnsonii* NCC533 [15] were used to identify BSH genes from *L*. *johnsonii* LB1 and *L*. *johnsonii* NCK88 contigs using BLASTp. All putative translated open reading frames were also screened to ensure no additional BSH genes were present in either LB1 or NCK88. Sequences were translated and aligned by ClustalW with MEGA6 [18]. Maximum Likelihood phylogenic reconstruction of the alignment was performed by bootstrapping with 100 replications based on the Le Gascuel 2008 model of amino acid substitution [17]. The amino acid substitution model was selected based on the lowest Bayesian Information Criterion (BIC) score of maximum likelihood fit from 56 different amino acid substitution models. Active site amino acids were identified with NCBI’s Conserved Domains Database search tool [51].

### BSH cloning

BSH genes identified for sequence comparisons (see previous section) in the genomes of *L*. *johnsonii* LB1, *L*. *johnsonii* NCK88, *L*. *johnsonii* ATCC33200, and *L*. *acidophilus* NCFM were amplified using primers containing the *Nco*I restriction recognition sequence and targeting the beginning and end of the coding sequences (Table B in S1 File). PCR amplicons were cloned into pSF-OXB12 (Oxford Genetics) using high efficiency DH5α competent cells (New England Biolabs) and proper insert orientation was confirmed by Sanger DNA sequencing at the Penn State Genomics Core Facility. The *NcoI* cloning site positions the ORF downstream of a Shine-Delgarno site encoded by pSF-OXB12, and changes the second codon and active site residue from “TGT” to “GTG”. The original ORF sequences were restored using New England Biolabs’ site directed mutagenesis kit, and the final constructs were confirmed by DNA sequencing and transformed into *E*. *coli* C600.

### BSH activity assay

For *Lactobacillus* whole cell assays, cells were centrifuged and washed twice with sterile PBS, pH 7.4, then resuspended in fresh media. T-β-MCA (Steraloids) was added to a concentration 500 nM, and cultures were incubated at 37°C for 90 mins. This concentration was chosen as it provided visible peaks by GCMS analysis and also allowed us to visualize concentration changes over time (see UPLC-ESI-QTOFMS analysis below). Cells were precipitated by centrifugation and 100 μL of supernatant was added directly to 100 μL of ice cold methanol and stored at -80°C for analysis by UPLC-ESI-QTOFMS. Cell lysate assays were used to assess BSH substrate specificity in transgenic *E*. *coli* C600 strains and were performed by washing overnight cultures twice in sterile PBS, pH 7.4, and resuspending the cells in 3 mM sodium acetate buffer, pH 5.2. Cells were lysed by vortexing with 0.1 mm glass beads (Mo Bio Laboratories, Inc.) according to the manufacturer’s instructions. Total protein was measured by Bradford assay and normalized to 0.100 mg/mL in 100 μL of buffer before adding a concentrated master solution of T-β-MCA, TCA, TDCA, TCDCA, GCA, and GCDCA (Sigma) to achieve a final concentration of 500 nM. Cell lysates were incubated at 37°C for 90 mins, quenched with 100 μL of ice cold methanol and stored at -80°C for analysis by UPLC-ESI-QTOFMS.

### UPLC-ESI-QTOFMS analysis

UPLC-ESI-QTOFMS analysis was performed in positive and negative mode with a G2S QTOFMS (Waters Corp), which was operated in full-scan mode at m/z 100–1,000. The liquid chromatography system was an ACQUITY UPLC (Waters Corp.) consisting of a reverse-phase 2.1x-50 mm ACQUITY UPLC BEH C18 1.7 μm column (Waters Corp.) with a gradient mobile phase comprising 0.1% formic-acid solution (A) and acetonitrile containing 0.1% formic acid solution (B). The gradient was maintained at 100% A for 0.5 min, increased to 100% B over the next 7.5 min and returned to 100% A in last 2 min. Nitrogen was used as both cone gas (50 l h^-1^) and desolvation gas (600 l h^-1^). Source temperature and desolvation temperature were set at 120°C and 350°C, respectively. The capillary voltage and cone voltage were 3,000 and 20 V, respectively. Quantification of bile-acid composition was determined by comparison against bile acid standards, and positive activity was defined as a 20% reduction in substrate concentration over the course of the assay in three independent experiments. We chose to represent data in this manner due to the high standard deviation observed between replicates, and a 20% reduction provided a clear separation of strains with BSH activity from those lacking it. As the hypothesis driving this manuscript was that T-β-MCA active lactobacilli colonize the caecum of mice in our C57BL/6J colony, and we were not attempting to make conclusions based upon enzyme specific activity, we did not attempt to identify the reason(s) behind the variation in our assay. Data was analyzed with Peakview^TM^ software version 1.1.0.0 (AB SCIEX).

### Predictive modeling

BSH protein structures were predicted from amino acid sequences using I-TASSER [52]. PDB coordinate files for each conjugated bile acid substrate were generated from Isomeric SMILES in the PubChem database using the National Cancer Institute’s Online SMILES Translator and Structure File Generator. Bile acid and BSH PDB files were merged using the crystal structure of the *C*. *perfringens* BSH bound to TDCA as a guide in Coot [19,53]. This BSH was chosen as it was the most closely related for which a structure was available. Finally, each predicted BSH/Bile acid complex was energetically minimized using the YASARA Energy Minimization Server [54]. Distance measurements for hydrogen bonding and hydrophobic interactions as well as figures for publication were generated with PyMOL (Schrödinger). CABS-flex was used to simulate protein structure fluctuations and assess the range of motion of loop structures within BSH protein structures [21].

## Supporting information

S1 File Supporting information. (DOCX)
