# Supplementary material for: Identification of a mouse Lactobacillus johnsonii strain with deconjugase activity against the FXR antagonist T-β-MCA
Source: PLoS One. 2017 Sep 14;12(9):e0183564. doi: 10.1371/journal.pone.0183564 (PMC5598929; doi:10.1371/journal.pone.0183564)
Supplement: S1 File — (DOCX) [file pone.0183564.s001.docx]

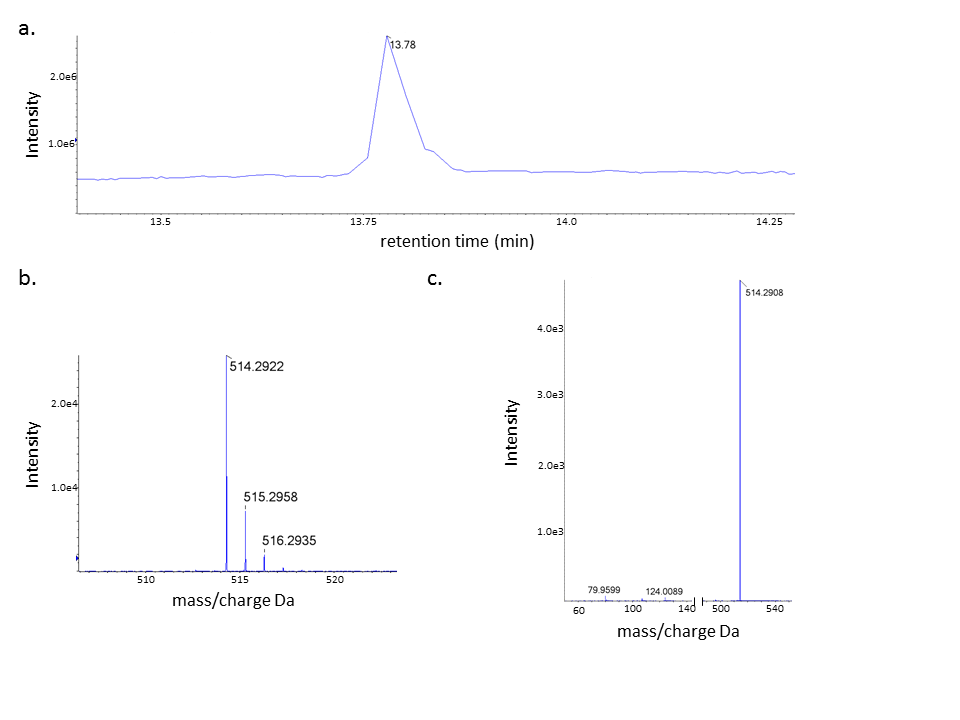


**Fig A. UPLC-ESI-QTOFMS identification of T-β-MCA.** A) The extracted ion chromatograph at m/z 514.2844 +/- 0.0025 Da with the corresponding retention time for T-β-MCA. B) MS peaks for 13.755 to 13.831 mins. C) MS-MS peaks for 13.765 mins. Images were generated with Peakview^TM^ software version 1.1.0.0 (AB SCIEX).


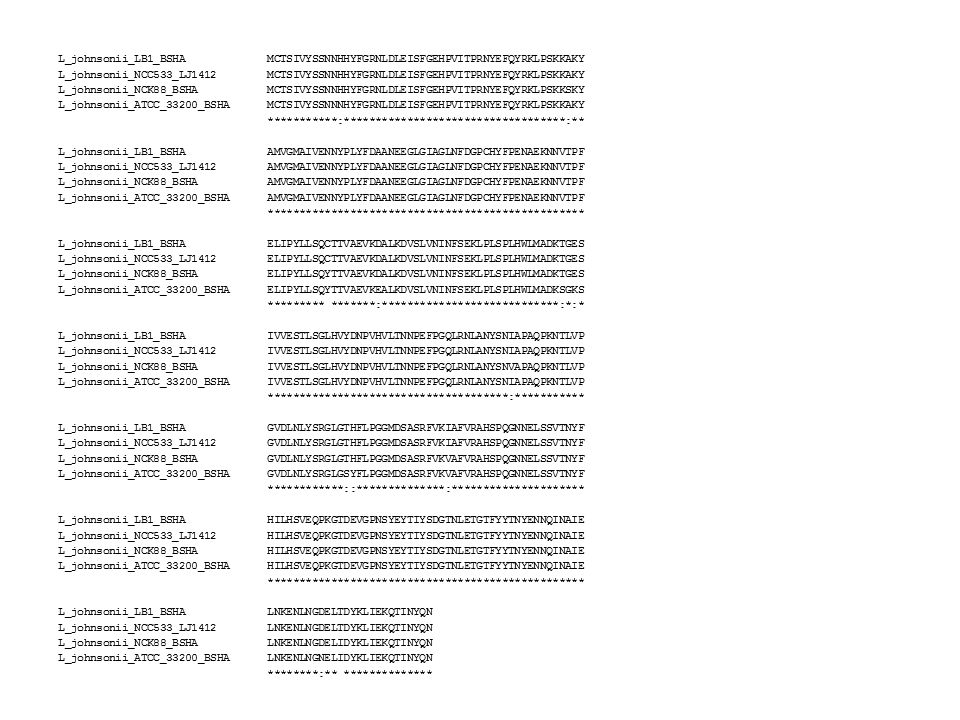


**Fig B. ClustalW multiple sequence alignment of BSHA homologs from *L. johnsonii* strains LB1, NCC533, NCK88, and ATCC 33200.** Conservation of residues among the four strains is indicated with an asterisk (*), and conservation among three strains is indicated with a colon (:).


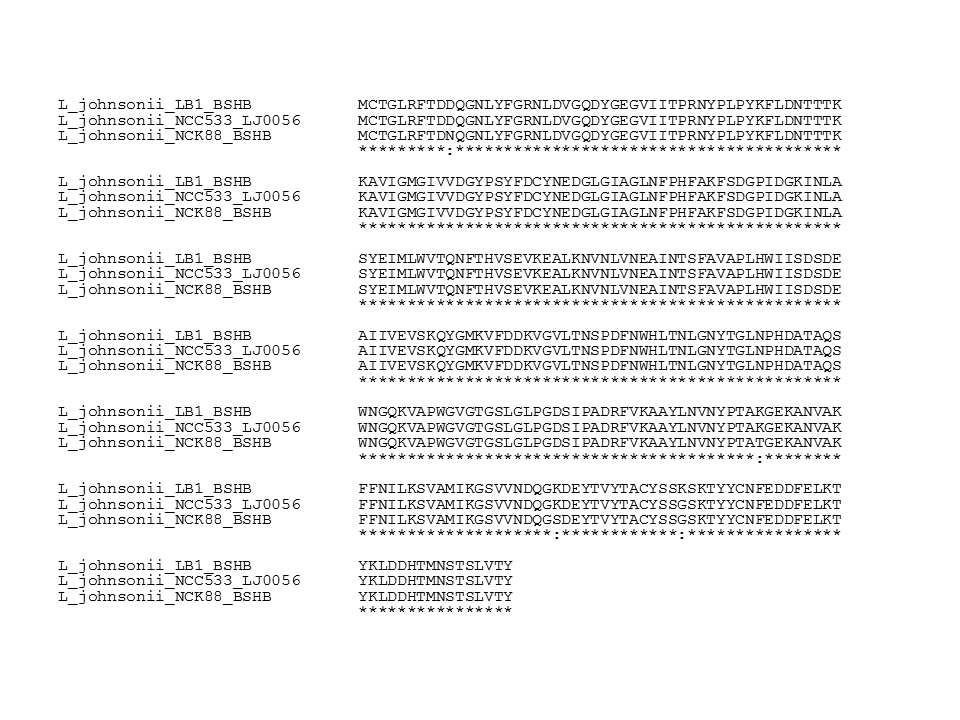


**Fig C. ClustalW multiple sequence alignment of BSHB homologs from *L. johnsonii* strains LB1, NCC533, and NCK88.** Conservation of residues among the three strains is indicated with an asterisk (*) and conservation among two strains is indicated with a colon (:).


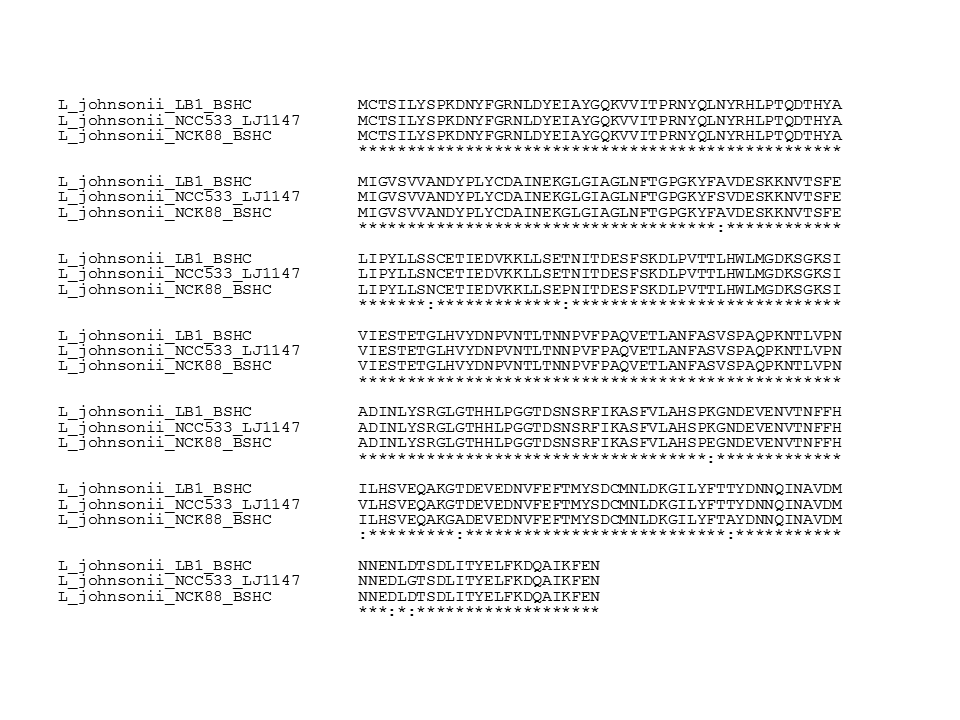


**Fig D. ClustalW multiple sequence alignment of BSHC homologs from *L. johnsonii* strains LB1, NCC533, and NCK88.** Conservation of residues among the three strains is indicated with an asterisk (*) and conservation among two strains is indicated with a colon (:).


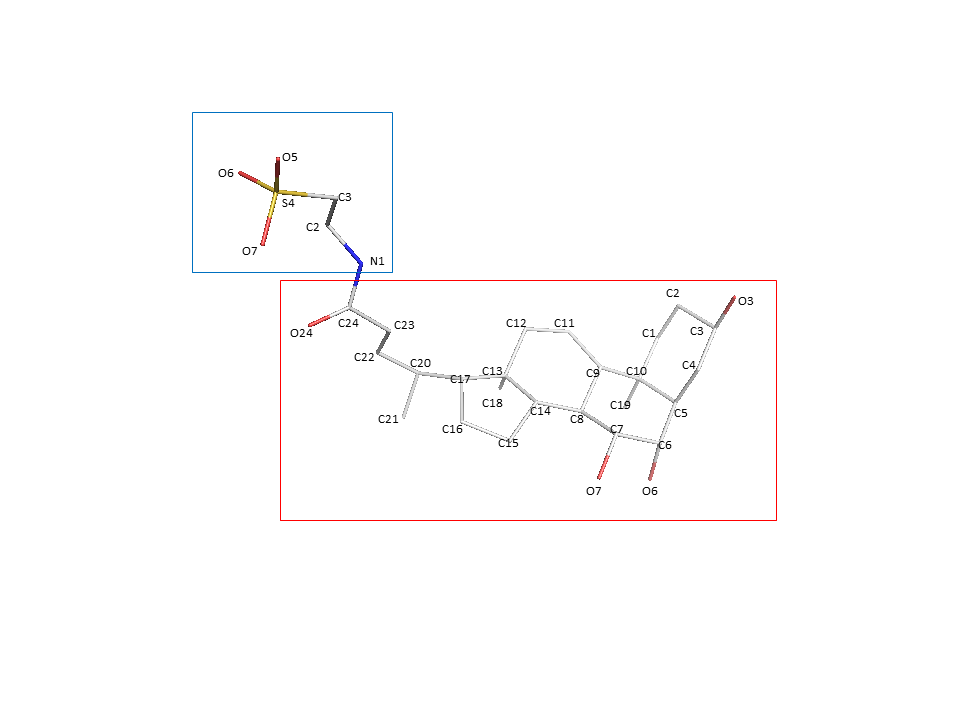


**Fig E. Labeling designations for T-β-MCA**

**Table A: Distance measurements between active site residues and T-β-MCA in models of *L. johnsonii* BSHs**

| **NCK88 BSHB** | **β-MCA** | **Distance (Å)** | **LJ LB1 BSHB** | **β-MCA** | **Distance (Å)** | **NCK88 BSHC** | **β-MCA** | **Distance (Å)** | **LJ LB1 BSHC** | **β-MCA** | **Distance (Å)** |
| --- | --- | --- | --- | --- | --- | --- | --- | --- | --- | --- | --- |
| Van der Waals Interactions (max 4.0A) | | | | | | | | | | | |
| C20LEU | C24 | 3.40 | C58ILE | C11 | 3.65 | C58ALA | C19 | 3.68 | C24TYR | C2 | 3.78 |
| C26TYR | C2 | 3.87 | C58ILE | C12 | 3.93 | C136THR | C15 | 3.78 | C63LEU | C18 | 3.63 |
| C136PHE | C3 | 3.65 | C67PHE | C21 | 3.62 | C65CYS | C21 | 3.52 | C63LEU | C18 | 3.66 |
| C136PHE | C3 | 3.98 | C67PHE | C21 | 3.66 | C65CYS | C21 | 3.54 | C65CYS | C20 | 3.63 |
| C136PHE | C4 | 3.86 | C136PHE | C3 | 3.59 | C134PRO | C4 | 3.83 | C65CYS | C21 | 3.67 |
| C136PHE | C4 | 3.99 | C136PHE | C4 | 3.85 | C136THR | C16 | 3.97 | C65CYS | C21 | 3.6 |
| C67PHE | C18 | 3.87 | C136PHE | C5 | 3.89 | C138LEU | C16 | 3.92 | C65CYS | C22 | 3.9 |
| C67PHE | C20 | 3.97 | C139ALA | C14 | 3.91 | C63LEU | C18 | 3.81 | C136THR | C16 | 3.87 |
| C67PHE | C20 | 3.90 | C139ALA | C15 | 3.92 | C63LEU | C19 | 3.96 | C136THR | C15 | 3.8 |
| C67PHE | C12 | 3.66 | C66TYR | C21 | 3.92 | C63LEU | C18 | 3.65 | C102LEU | C21 | 3.87 |
| C67PHE | C12 | 3.69 | C141LEU | C23 | 3.91 |  |  |  | C20TYR | N26 | 3.65 |
| C67PHE | C21 | 3.72 | C60VAL | C19 | 3.90 |  |  |  | C133LEU | C4 | 3.98 |
| C141LEU | C23 | 3.92 |  |  |  |  |  |  | C138LEU | C16 | 3.86 |
|  |  |  |  |  |  |  |  |  | C56VAL | C11 | 4.00 |
|  |  |  |  |  |  |  |  |  | C58ALA | C19 | 3.80 |
|  |  |  |  |  |  |  |  |  |  |  |  |
| **NCK88 BSHB** | **Taurine** | **Distance (Å)** | **LJ LB1 BSHB** | **Taurine** | **Distance (Å)** | **NCK88 BSHC** | **Taurine** | **Distance (Å)** | **LJ LB1 BSHC** | **Taurine** | **Distance (Å)** |
| H-Bonding (max 3.4A) | | | | | | | | | | | |
| O21ASN | O5 | 3.12 | OH26TYR | N1 | 3.10 | O19ASN | S4 | 3.18 | O19ASN | O5 | 2.82 |
| N21ASN | O5 | 2.94 | N21ASP | O5 | 2.95 | O19ASN | O5 | 2.85 | S2CYS | O5 | 3.12 |
| S2CYS | O6 | 3.09 | N18ARG | O5 | 3.13 | N19ASN | O5 | 3.17 | N79ASN | O7 | 3.13 |
| N81ASN | O6 | 3.05 | S2CYS | O6 | 3.30 | S2CYS | O5 | 2.91 |  |  |  |
|  |  |  | N81ASN | O7 | 3.08 | N79ASN | O6 | 3.03 |  |  |  |
|  |  |  |  |  |  | S2CYS | O6 | 3.12 |  |  |  |
|  |  |  |  |  |  | N2CYS | O7 | 3.17 |  |  |  |

**Table B: Primer sequences for BSH cloning, site directed mutagenesis, and sequencing**

| **Primers for BSH cloning into pSF-OXB12** | |
| --- | --- |
| **Primer Name/Target** | **Sequence (5’-3’)*** |
| LB1 BSHA FWD | ATATACTCGAGATGTGTACCTCAATTGTTTATAGT |
| LB1 BSHA REV | ATATACTCGAGTGTTGAACAGGAAGAAGCTTTA |
| LB1 BSHB FWD | ATATACTCGAGATGTGTACTGGTTTAAGATTCACAG |
| LB1 BSHB REV | ATATACTCGAGATCGAGCGGTTGACGGTATT |
| LB1 BSHC FWD | ATATACTCGAGATGTGTACATCAATTTTATATAGTCCA |
| LB1 BSHC REV | ATATACTCGAGTGATCAAATCATGTCCAGTA |
| NCK88 BSHB FWD | ATATACTCGAGATGTGTACTGGTTTAAGATTCACAG |
| NCK88 BSHB REV | ATATACTCGAGCCGGTATTGCGATATCAGGCT |
| NCK88 BSHC FWD | ATATACTCGAGATGTGTACATCAATTTTATATAGTCCA |
| NCK88 BSHC REV | ATATACTCGAGGAATGATCAAATCATGTCCAGT |
| ATCC 33200 BSHA FWD | ATATACTCGAGATGTGTACCTCAATTGTTTAT |
| ATCC 33200 BSHA REV | ATATACTCGAGCAGATATCTTCTATCTGGCAA |
| **Primers for Site Directed Mutagenesis** | |
| **Primer Name/Target** | **Sequence (5’-3’)** |
| SDM pSF-OXB12 (used for all constructs) | GGTGGGTACCTCCTTTGA |
| SDM LB1 BSHA | ATGTGTACCTCAATTGTTTATAGTTC |
| SDM LB1 BSHB | ATGTGTACTGGTTTAAGATTCAC |
| SDM LB1 BSHC | ATGTGTACATCAATTTTATATAGTCC |
| SDM NCK88 BSHB | ATGTGTACTGGTTTAAGATTCAC |
| SDM NCK88 BSHC | ATGTGTACATCAATTTTATATAGTCC |
| SDM ATCC 33200 BSHA | ATGTGTACCTCAATTGTTTATAGTTC |
| **Sequencing primers** | |
| **Primer Name/Target** | **Sequence (5’-3’)** |
| pSF-OXB12 FWD | GATCTTTGTCGATCCTACCATCC |
| pSF-OXB12 REV | CGCTGTATCTCAGTCAGTCAAG |

*The XhoI site is underline
